# The Role of Hemoglobin A1C in Diabetes Screening and Diabetic Retinopathy

**DOI:** 10.3390/jcm10214947

**Published:** 2021-10-26

**Authors:** Maria Mercedes Chang Villacreses, Rudruidee Karnchanasorn, Horng-Yih Ou, Raynald Samoa, Lee-Ming Chuang, Ken C. Chiu

**Affiliations:** 1 Department of Clinical Diabetes, Endocrinology and Metabolism, City of Hope National Medical Center, Duarte, CA 91010, USA; mchangvillacreses@coh.org (M.M.C.V.); rsamoa@coh.org (R.S.); 2 Division of Endocrinology, Department of Medicine, University of Kansas Medical Center, Kansas City, KS 66160, USA; rudruidee@gmail.com; 3 Division of Endocrinology and Metabolism, Department of Internal Medicine, National Cheng-Kung University Medical College and Hospital, Tainan 704, Taiwan; wahoryi@mail.ncku.edu.tw; 4 Department of Internal Medicine, National Taiwan University Hospital, Taipei 102, Taiwan; leeming@ntu.edu.tw; 5 Institute of Clinical Medicine, College of Medicine, National Taiwan University, Taipei 102, Taiwan; 6 Division of Endocrinology, Metabolism and Nutrition, Department of Internal Medicine, Harbor-UCLA Medical Center, Torrance, CA 90502, USA

**Keywords:** diabetes mellitus, diagnosis, sensitivity, specificity, retinopathy

## Abstract

Hemoglobin A1C (A1C) is used in various settings. Its performance has not been evaluated systemically. We compared A1C in diagnosis of diabetes with fasting plasma glucose (FPG) and 2-h postchallenged plasma glucose (2hPG) parameters in a cross-sectional cohort in the United Stated. Adult subjects (≥20 years) were identified from the National Health and Nutrition Examination Survey 2005–2016 without a history of diabetes who had BMI, A1C, FPG, and 2hPG (*n* = 10,416). For comparisons, we calculated the sample weighted prevalence, sensitivity, specificity, positive predictive value (PPV), and negative predictive value (NPV) with subgroup analyses. For the retinopathy study, diabetic subjects with established diabetes who responded to the question of diabetic retinopathy were evaluated (*n* = 3907). Compared to the FPG/2hPG criteria, A1C ≥ 48 mmol/mol (6.5%) had a low sensitivity at 25.90%, with specificity 99.70%, PPV 84.70%, and NPV 95.70%. Subgroup analyses revealed a lower sensitivity in males (24.52%); the lowest in non-Hispanic White (21.35%), in the third decade (14.32%), and in the BMI < 22.50 kg/m^2^ group (7.21%). The prevalence of self-reported diabetic retinopathy increased drastically with an inflection point at A1C 48 mmol/mol (6.5%) from 11.52% to 18.32% (*p* < 0.0001). A1C ≥ 48 mmol/mol (6.5%) should be cautiously used to diagnose diabetes in certain subgroups due to very low sensitivity in certain groups. With the confirmation of the association of increasing self-reported diabetic retinopathy with A1C ≥ 48 mmol/mol (6.5%), the current A1C cutoff is an acceptable value with the understanding of especially low sensitivity in certain subgroups.

## 1. Introduction

The prevalence of diabetes mellitus (DM) has been steadily increasing since 1999 [1]. According to the National Diabetes Statistics Report 2020 from the Centers of Disease Control and Prevention (CDC), the estimated prevalence of DM was 13.0% of the US adult population (34.1 million), of which 7.3 million people (21.4%) of people with DM were still undiagnosed [2]. Interestingly, the prevalence of total DM and diagnosed DM increased in parallel while the prevalence of undiagnosed DM decreased since 2010. Although no definite explanation for the decreasing prevalence of undiagnosed DM is available, the improvement in the diagnosis of DM could play a role. In 2009 hemoglobin A1C (A1C) was adapted to be used in the diagnosis of DM [3]. Early diagnosis and intervention of diabetes is highly recommended since serious diabetic complications is preventable through early intervention [4,5]. 

The diagnosis of DM is mainly based on fasting plasma glucose (FPG), 2-h postchallenged plasma glucose (2hPG), or A1C. In 1979, based on the National Diabetes Data Group, the diagnosis of DM was established using FPG of ≥7.8 mmol/L (≥140 mg/dL), or a 2hPG level of ≥11.1 mmol/L (≥200 mg/dL). With the discovery of glucokinase deficit as a cause of DM [6] and recognition of mildly elevated fasting plasma glucose, the FPG threshold was decreased to ≥7.0 mmol/L (≥126 mg/dL) in 1997 [7]. Since then, the popularity of FPG in the diagnosis of DM increased while FPG had a lower sensitivity than 2hPG in the diagnosis of DM [8], however, fasting was still required. Thus, the alternative diagnostic criterium for DM without the need of preparation was in demand, which is A1C. It was originally discovered in 1968 [9], and subsequently proposed as a clinical tool for the management of patients with DM [10]. The use of A1C in the diagnosis of DM was only established after a standardization of A1C measurement and adapted by the American Diabetes Association (ADA) in 2009 [3] and by the World Health Organization in 2011 [11]. Due to no preparation required and being able to be performed at any time, A1C became the preferred method in diagnosis of DM in general practice [12,13].

Despite ADA stating that DM may be diagnosed by FPG, 2hPG, and A1C with no preference [14], A1C has become widely used in various clinical and research settings, such as defining the prevalence of DM [15,16]. Although the low sensitivity of A1C in diagnosis of DM has been noted [8], the factors that may affect the performance of A1C in the diagnosis has not been well described. In this study, we examined the impact of gender, age, race/ethnicity, and body mass index (BMI) on the performance of A1C for disease diagnosis in a cross-sectional cohort in the United States of America. We also investigated the relationship of diabetic retinopathy with A1C which was originally used to establish the current A1c criterion.

## 2. Materials and Methods

### 2.1. Study Design

The National Health and Nutrition Examination Survey (NHANES) has been conducted by the National Center for Health Statistics of the CDC since 1960’s in the United Stated of America. It is a cross-sectional national survey which occurs every 2 years in a representative population in the United States. The study has been reviewed and approved by National Center for Health Statistics Research Ethics Review Board since 1999 (last protocol code #2018-01, approved on 26 October 2017). Informed consent was obtained from the participants at the time of enrollment. Before releasing, the records were anonymized and de-identified. Only de-identified data from this survey was included in this study for analysis which is exempt from the federal regulations for the protection of human research participants. The data is available to public at the NHANES website. This is a cross-section study design to examine the performance of the current A1C criterion for diabetes in comparison to FPG and 2hPG in a representative population in the United States with subgroup analyses.

### 2.2. Studied Subjects

All the data was extracted from the NHANES databases from the years 2005 to 2016, since the oral glucose tolerance tests were reintroduced in 2005. From a total of 60,936 participants, we included those who were at least 20 years old and possessed all measurements: BMI, A1C, FPG, and 2hPG. After excluding those patients with a history of DM, the sample size was reduced to 10,416 individuals without a prior diagnosis of diabetes (Table 1).

For the retinopathy study, we only included the subjects who identified themselves to have DM (*n* = 4338) from the NHANES 2005–2016 cohort. Of the 4338 participants, only the subjects who responded to the retinopathy question and had a measurement of A1C (*n* = 3907) were included. Since the current diagnostic criteria of DM were based on the inflection of the prevalence of diabetic retinopathy [7], the relationship of A1C with other microvascular complications, such as nephropathy and neuropathy, were not examined in this study.

### 2.3. Assessments

Age in years at the time of the screening was reported for participants and gender was defined as male or female based on self-report. BMI was calculated as weight in kilograms divided by height in meters squared. Race/ethnicity was self-reported by participants and categorized into Mexican American, Other Hispanic, non-Hispanic White, non-Hispanic Black, and other which included non-Hispanic race and non-Hispanic multiracial participants.

### 2.4. Definition of Diabetes Mellitus by FPG, 2hPG, and A1C

We adapted the established criteria by the ADA in this study [13]. DM was defined as either A1C ≥ 48 mmol/mol (≥6.5%), FPG ≥ 7.0 mmol/L (≥126 mg/dL), or 2hPG ≥ 11.1 mmol/L (≥200 mg/dL) during a standard oral glucose tolerance test. 

### 2.5. Laboratory Methods

Plasma glucose was analyzed by using hexokinase method. Due to different laboratory instruments used to measure plasma glucose in 2005–2006, a regression equation was applied to align plasma glucose concentrations obtained in 2005–2006 as recommended by the NHANES. FPG samples were obtained in the morning session after a 9-h fast. 2hPG samples were obtained at 2 h after the initial venipuncture for FPG followed by a drink 75 g of glucose solution. A1C was analyzed by using the HPLC analytical columns. Based on the recommendation of National Glycohemoglobin Standardization Program and NHANES, no regression equations were used for A1C analysis. 

### 2.6. Statistical Analysis

The proportional variables were compared using chi-square tests. Continuous variables were compared using Student’s *t*-tests or ANOVA tests when it is appropriate. Proportional variables were presented as count with percent, while continuous variables were presented as mean with standard deviation. The NHANES samples are based on a complex, multistage, and probability sampling design. To accommodate unequal probabilities of sample selection due to complex sample design and oversampling of certain subgroups, sampling weights were adjusted in all specified analyses as recommended by NHANES. The sensitivity and specificity of A1C in the diagnosis of diabetes in comparison to FPG and/or 2hPG were calculated with 95% confidence intervals along with positive and negative predictive values (PPV and NPV, respectively). Correlations of A1C with FPG and 2hPG were investigated using the least squares linear regression analyses. Sample weighted results were presented unless otherwise specified. All the analyses were conducted in SYSTAT 13, Systat Software, Inc., Chicago, Illinois, USA. A nominal *p* value of less than 0.05 was considered significant.

## 3. Results

### 3.1. Clinical Characteristics

This study evaluated 10,416 adult subjects without a prior history of DM. Their clinical characteristics were described in Table 1. To increase the reliability and precision of estimates of health status indicators for these population subgroups, oversampling is conducted at various stages of NHANES. Between 1999 and 2006, oversampling was performed for Mexican American and between 2007 and 2010, oversampling was performed for all Hispanic persons. Since 2011, oversampling was performed for Asians. The distribution of race/ethnicity reflected the oversampling scheme. The weighted prevalence of undiagnosed DM defined by any of FPG, 2hPG, and A1C in this cohort was 5.98% (95% CI: 5.97–5.98).

### 3.2. Comparison of Diagnosis of Diabetes Mellitus Using the Plasma Glucose and A1C Criteria

Diagnosis of DM based on the FPG/2hPG vs. A1C criteria was evaluated (Table 2a). With sample weighted analysis, 5.70% of participants were diabetic based on FPG/2hPG while only 1.75% of participants were diabetic based on A1C (*p* < 0.0001). The A1C criterion had a fairly-low sensitivity of 25.90%, and missed 71.40% of diabetic participants who were defined to be diabetic using the FPG/2hPG criteria. The current A1C cutoff defined DM in an additional 0.30% of non-diabetic participants based on the FPG/2hPG criteria. 

### 3.3. Impact of Gender on the Diagnosis of Diabetes Mellitus Using the Plasma Glucose and A1C Criteria

There was a significant difference in sensitivity (*p* < 0.0001) using the A1C criterion between males (27.31%) and females (24.52%) as described in Table 2b. A higher sensitivity for male participants compared to female participants could be due to the higher prevalence of undiagnosed DM in male participants (males: 6.17% vs. females: 5.81%; *p* < 0.0001). 

### 3.4. Impact of Race/Ethnicity on the Diagnosis of Diabetes Mellitus Using the Plasma Glucose and A1C Criteria

When the data was stratified by race/ethnicity (Table 2c), the sensitivity of the current A1C cutoff in detecting DM was not great at all in all racial/ethnic groups, as compared to the FPG/2hPG criteria. The lowest sensitivity was noted in non-Hispanic White (21.35%), while the highest sensitivity was noted in other race/ethnicity (41.55%), which was almost identical as non-Hispanic Black (41.27%).

### 3.5. Impact of Age on the Diagnosis of Diabetes Mellitus Using the Plasma Glucose and A1C Criteria

Age was stratified by decade (Table 2d), and the lowest sensitivity was noted in the third decade (20–29 years old; 14.32%), while the highest sensitivity was noted in the fourth decade (30–39 years old; 36.21%). The trend of sensitivity could not be explained by the weighted prevalence of undiagnosed DM, the lowest in the third decade (1.08%) while the highest in the sixth decade (50–59 years old; 6.76%). Of note, 26.94% of participants in the third decade had BMI < 22.5 kg/m^2^ in contrast to 12.00–15.98% in other age groups, while it was 14.75% in the fourth decade. Thus, the effect of BMI could not be explained the trend of sensitivity, either. 

### 3.6. Impact of Body Mass Index on the Diagnosis of Diabetes Mellitus Using the Plasma Glucose and A1C Criteria

We divided BMI by 2.50 kg/m^2^ from 22.50 to 37.50 kg/m^2^, as shown in Table 2e. The lowest sensitivity (7.21%) was noted in the leanest group (<22.50 kg/m^2^), while the highest sensitivity (46.02%) was observed in the most obese group (≥37.50 kg/m^2^, Table 2e). Although the weighted prevalence of undiagnosed DM increased gradually from the leanest group (1.96%) to the most obese group (12.66%), the sensitivity did fluctuate throughout the course. Furthermore, the trend of sensitivity by BMI subgroups could not be explained by the differences in the racial/ethnic groups and age groups. 

### 3.7. Clinical Characteristics of Defined by Different Diagnostic Criteria of Diabetes

Table 3 showed that clinical characteristics differed significantly among three diabetic subgroups (*p* < 0.0001), based on different diagnostic criteria. However, there were no difference in age between DM defined using FPG/2hPG only but not A1c and using A1C only but not FPG/2hPG; and in BMI between DM defined using both FPG/2hPG and A1c and using A1C only but not FPG/2hPG. The diabetic subjects defined using both the A1C and FPG/2hPG criteria, 79.22% had both elevated FPG and 2hPG, while the diabetic subjects defined using FGP/2hPG but not A1C, only 18.90% had elevated both FPG and 2hPG.

### 3.8. Alternative A1C Criteria for the Diagnosis of Diabetes Mellitus

Due to the low sensitivity of the current A1C cutoff as compared to the FPG/2hPG criteria, we sought for alternative A1C criteria. The correlation between A1C and FPG [A1C = 3.1708 + (0.0225 × FPG), A1c in % and FPG in mg/dL] was noted (r = 0.6731, *p* < 0.0001). FPG of 126 mg/dL (7.0 mmol/L) intercepted A1C at 6.0% (42 mmol/mol). Similarly, the correlation of between A1C and 2hPG [A1C = 4.6973 + (0.0062 × 2hPG), A1C in % and 2hPG in mg/dL) was also noted (r = 0.5550, *p* < 0.0001). 2hPG of 200 mg/dL (11.1 mmol/L) intercepted A1C at 5.9% (41 mmol/mol). Accordingly, we constructed the sample weighted prevalence of undiagnosed DM, sensitivity, specificity, PPV, NPV, and the area under receiver operating characteristic (ROC) curve for each A1C criterion from 5.5% (37 mmol/mol) to 6.4% (46 mmol/mol) with an increment of 0.1% (Table 4). With decreasing A1C, the sample weighted prevalence of undiagnosed DM, sensitivity, NPV, and the area under the ROC curve increased while specificity and PPV decreased. 

### 3.9. Diabetic Retinopathy in Established Diabetic Subjects

In this cohort the weighted prevalence of established DM was 6.79% (*n* = 4338). Within the established DM cohort, 2681 subjects (62.65%) reported to have dilated eye examination within the past 12 months before the survey and 604 subjects (14.94%) within the past 13 to 24 months. Among the established diabetic subjects, 78 subjects (1.33%) failed to provide proper responses to diabetic retinopathy and 920 subjects (19.00%) reported to have diabetic retinopathy. To investigate the relationship between diabetic retinopathy and A1C, we only included those with established DM, who responded to the retinopathy question, and had available A1C results (*n* = 3907). To reduce sampling bias from small sample size and improve granularity of A1C, we divided the subjects into 18 categories based on A1C with a target of 250–300 subjects in each category and the smallest increment A1C if possible, especially between 6.0% (42 mmol/mol) and 8.0% (64 mmol/mol) as showed in Table 5. The weighted prevalence was fairly-stable for A1C ≤ 6.2% (44 mmol/mol), and increased drastically for A1C ≥ 6.5% (48 mmol/mol).

## 4. Discussion

In this study, we demonstrate that the current A1C cutoff has a significant tendency of underdiagnosing DM with an exceptionally low and significant variable sensitivity, such as the lowest sensitivity, 7.21% in subjects with BMI < 22.5 kg/m^2^ by BMI category, 14.32% in the third decade of life (20–29 years old) by age category, and 21.35% in non-Hispanic Whites by racial/ethnic category, 24.52% in females by gender category. Even the highest sensitivity was 46.02% in the most obese category (BMI ≥ 37.5 kg/m^2^) which was still less than 50%. Thus, the current criterion of A1C ≥ 48 mmol/mol (≥6.5%) has an exceptionally low sensitivity in the diagnosis of DM by missing more than 50% of DM defined by the gold standard which is based on the FPG and/or 2hPG criteria. 

Although it is not recommended, A1C is the preferred method in the diagnosis of DM as no preparation is required and can be obtained at any time as compared to FPG and 2hPG as reflected in the NHANES. In the NHANES 2005–2016, A1C was obtained in 71.71% of participants. In contrast, due to the requirement additional preparation and additional burden to both participants and survey conducting personnel, FPG and 2hPG were only obtained from a subsample of participants who were examined in the morning session after fasting, 38.44% and 29.91%, respectively. Since A1C only picked up 25.90% of undiagnosed diabetic adult participants diagnosed using FPG and/or 2hPG, substantial amount, 74.10%, of undiagnosed adult participants would be missed using A1C alone. Our observations are consistent with previous reports of low sensitivity of the current A1C cutoff [7,16]. Thus, it is a significant public health and clinical issue by missing diagnosis of diabetes exclusively based on A1C. Furthermore, there is a miss opportunity for early intervention of diabetes and preventing the development of serious diabetic complications.

To further explore the low sensitivity of the current A1C criterion, we compared the three groups of clinical characteristics of previously undiagnosed diabetic subjects based on different diagnostic criteria: FPG/2hPG but not A1C, both A1C and FPG/2hPG, and A1C but not FPG/2hPG (Table 3). As compared to those with DM defined by both FPG/2hPG and A1C, the diabetic subjects defined with FPG/2hPG but not A1C were older (62 vs. 56 years old, *p* = 0.004), more female (51.57% vs. 47.95%, *p* < 0.0001), less obese (BMI 30.19 vs. 33.45 kg/m^2^, *p* < 0.0001), and significant difference in racial/ethnic distribution (*p* < 0.0001). These results are consistent with the results of the subgroup analyses (Table 2b–e). When compared to those with DM defined by both FPG/2hPG and A1C, the diabetic subjects defined A1C but not FPG/2hPG were at odds with the results of the subgroup analyses (Table 2b–e) in respective to age, gender distribution, and BMI while with way more African Americans (47.78%), which could be related to the issue with disparities in A1C levels of African American with other race/ethnicity [17,18,19]. When comparing to the group with DM defined using both FPG/2hPG and A1c, the group with DM defined using FPG/2hPG but not A1c had more isolated elevated FPG and isolated elevated 2hPG while less elevated both FPG and 2hPG (*p* < 0.0001). These results indicate that current A1C ≥ 6.5% criterion has a better concordance in the more severe diabetic subjects with significantly elevated both FPG and 2hPG, but not in the mild diabetic patients with isolated elevation of FPG or 2hPG (Table 3). Thus, the current A1C ≥ 6.5% criterion is too stringent as compared to the current gold standard FPG/2hPG criteria. 

Many argue that A1C cutoff criteria should be revised to obtain a better sensitivity, and several cutoff values of A1C have been proposed [8,20,21,22,23,24,25]. Based on the established relationship between A1C and blood glucose [24], the estimated average glucose concentration is 7.8 mmol/L (140 mg/dL) based on A1C of 48 mmol/mol (6.5%). From the A1C-Derived Average Glucose study [26,27] based on 470 diabetic patients, mean fasting glucose was 7.9 mmol/L (95% CI: 7.5–8.3) for A1C between 48–53 mmol/mol (6.5–7.0%). Based on the observed correlation between A1C and FPG [A1C = 3.1708 + (0.0225 × FPG), A1c in % and FPG in mg/dL), r = 0.6731, *p* < 0.0001] in the present study, the calculated FPG concentration is 8.2 mmol/L (148 mg/dL). Clearly, A1C of 48 mmol/mol (6.5%) is indicative for FPG way higher than 7.0 mmol/L (126 mg/dL), which is diagnostic criterion of diabetes by FPG. Thus, a lower A1C cutoff for the diagnosis of diabetes than current 48 mmol/mol (6.5%) is highly suggested. 

It has been demonstrated that mean plasma glucose (MPG) concentrations, defined by the average of multiple measurements of glucose taken throughout the day, is highly correlated with A1C (r = 0.81–0.95) with a change in MPG of about 1.9 mmol/L (35 mg/dL) for each 10.9 mmol/mol (1%) change in A1C, while FPG is also correlated with A1C (r = 0.62–0.67) and postprandial plasma glucose (r = 0.22–0.56) [28]. These results are consistent with our observations in respective to the correlation of A1C with FPG (r = 0.6751) and 2hPG (r = 0.5550). However, averaged FPG, PPG, and MPG were used, while the NHANES data were from a single measurement of FPG and 2hPG. Based the correlation of FPG and 2hPG with A1C, the current FPG and 2hPG criteria intercept A1C at 41 mmol/mol and 42 mmol/mol (6.0% and 5.9%), receptively. Accordingly, we searched for the alternative A1C criterion from the current sample set as shown in Table 4. Thus, the criterion of A1C ≥ 41 mmol/mol (6.0%) could be an alternative criterion, which is in accordance with the inflection of the prevalence of diabetic retinopathy from the NHANES III [7], but not with the observations from an Egyptian population [29] and a Pima Indians population [30]. Based on A1C ≥ 41 mmol/mol (6.0%), the results of subgroup analyses were showed in Table 5. Overall, the sensitivity improves with more than 50% on most of subgroup analyses while PPVs decreased drastically to 19.81% in African American subjects, and 18.93% in the leanest subjects by BMI. Very low PPV is the concern when A1C cutoff is lowed to 41 mmol/mol (6.0%). In addition, with drastic decrease in PPV and relatively minor changes in the area under the ROC curve as shown in Table 4, lowing A1C cutoff may not be completely desirable. 

**Figure 1:**
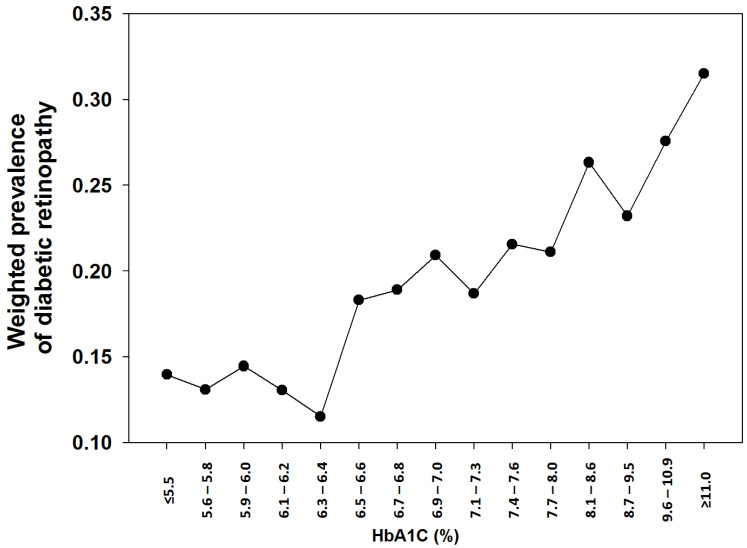
Weighted prevalence of diabetic retinopathy in subjects with established diabetes.

Although the current A1C cutoff was selected based on linear inflections of prevalence of diabetic retinopathy in three population studies [7,29,30] and supported by some studies [31,32], a lower A1C cutoff has been suggested by other studies for diabetic retinopathy [20,33,34]. To investigate the cross-sectional relationship of the prevalence of diabetic retinopathy and A1C in this cohort, we examined the prevalence of the self-reported retinopathy by the A1C categories in established diabetic participants (Table 5 and Figure 1), since ophthalmological examination, specifically retinal examination, was only available in a relatively small subset of the NHANES 2005–2006 and 2007–2008 only. The weighted prevalence of retinopathy was fairly-stable (13.05–14.46%) for A1C ≤ 44 mmol/mol (6.2%). A dip of the weighted prevalence was noted between the categories of 43–44 mmol/mol (6.1–6.2%) and 45–46 mmol/mol (6.3–6.4%) from 13.05% to 11.52% (*p* < 0.0001) which could be due to intrinsic sampling basis for the A1C category of 45–46 mmol/mol (6.3–6.4%). There was an incredibly significant increase of the weighted prevalence of retinopathy was noted on the category of A1C 48–49 mmol/mol (6.5–6.6%), when compared to the category of 45–46 mmol/mol (6.3–6.4%, 18.32% vs. 11.52%, respectively, *p* < 0.0001) and the category of 43–44 mmol/mol (6.1–6.2%, 18.32% vs. 13.05%, respectively, *p* < 0.0001). Of note, the self-reported prevalence of diabetic retinopathy (19.00%) is much lower than in previous publications in the US populations by retinal examination (28.5–40.3%) [34,35,36]. However, the prevalence of self-reported diabetic retinopathy before the inflection point is much higher in this cohort (11.52–14.46%) than the previously observed prevalence (<5% in the Pima Indians and NHANES III, and <10% in the Egyptians) used to define the current A1C diagnostic criterion [7]. The difference in the prevalence with the published studies could be from various factors; the self-reported retinopathy in this study while formal eye examinations in the published studies and the sampling bias as the subjects with the established retinopathy could have more frequent and regular eye examination. Since much less established diabetic subjects had FPG and even less 2hPG, we could not conduct a meaningful analysis based on FPG and 2hPG. Nevertheless, our observation supported an inflection point for diabetic retinopathy around A1C of 6.5% (48 mmol/mol) in the established diabetic patients based on the self-reported retinopathy by the participants. 

To our knowledge, the present study has the largest sample set (*n* = 10,416). Furthermore, our results are validated by weighted analyses for a complex sample design which consisted of oversampling of certain population subgroups to increase the reliability and precision of health status indicator estimates for these particular subgroups, and accommodating unequal probabilities of sample selection. With extensive subgroup analyses which has not been performed before, we identified the unexpected low sensitivity in each category. With weighted analyses, the results of our observation can be applied to the general population in the United States. However, since no retinal examination was performed in most of participants in the NHANES 2009–2016 and diabetes questionnaire was not administered in participants with undiagnosed diabetes, we are not able to investigate the relationship of clinical confirmed diabetic retinopathy with A1C, FPG, and 2hPG to validate the proposed A1C ≥ 42 mmol/mol (6.0%) criterion. In addition, the self-reported diabetic retinopathy may not accurately reflect the true underlying eye condition, which could be either understated or overstated depending on the conception of the responders. Furthermore, since the development of diabetic retinopathy may take years, a cross-sectional relationship based on a single A1C measurement will not truly reflect the impact of long-term glycemic control on the development of diabetic retinopathy. Nevertheless, a cross-sectional relationship of A1C ≥ 48 mmol/mol (≥6.5%) with an inflection point for the self-reported diabetic retinopathy is confirmed in this study. Since this is based on the survey within of the United States, further validation in other countries and regions are required. 

The strengths and limitations of this study are summarized as below. With the complex study design of the NHANES, we provide extended subgroups analyses which were not available before. With sample weighted analyses, the results of this report can be applied to the U.S. population. However, due to a cross-sectional study design, no cause-and-effect relationship can be defined. The diagnosis of diabetic retinopathy was based on the report of participants in this study, it clearly represents a bias that justifies the difference with the prevalence found in numerous other observational studies based on clinical examination of the fundus oculi. Since the current diagnostic criteria of DM were based on the inflection of the prevalence of diabetic retinopathy [6], the relationship of A1C with other microvascular complications, such nephropathy and neuropathy, was not examined in this study. With the self-reported diabetic retinopathy in this study, A1C of 48 mmol/mol (6.5%) as a diagnostic criterion uses a diagnosis of true diabetic retinopathy whose prevalence is certainly underestimated. Furthermore, early detection of diabetic retinopathy cannot be overemphasized, and it can be achieved through structured models of telemedicine [37,38], especially during the pandemic of coronavirus infection. In addition, the importance of a correct diagnosis of diabetic retinopathy allows not only to reduce the risk of eye damage and progression up to loss of vision, but also to identify subjects at high cardiovascular risk for early intervention as recently observed in a multicenter study [5].

## 5. Conclusions

Since diabetic complications can be prevented and/or reduced by early detection and intervention of DM, a screening test with a high sensitivity and no preparation required is highly desired and the current A1C criterion (≥6.5% or 48 mmol/mol) fails to meet the demand. However, with a lower A1C cutoff, the sensitivity improves while PPV decreases drastically and the area under ROC improves less than desired. With the confirmation of diabetic retinopathy, A1C ≥ 48 mmol/mol (≥6.5%) could be appropriate as a tradeoff for the screening of diabetes by A1C with some understanding of very low sensitivity in some subgroups of the population, such as third decade of life (14.3% in 20–29 years), lean (7.21% in BMI < 22.5 kg/m^2^), and non-Hispanic White (21.35%).

## Figures and Tables

**Table 1:** Clinical characteristics of the studied subjects without a prior diagnosis of diabetes.

		Mean (*n*)		STD (%)
*n*		10,416		
Age	Years	48	±	17
Gender	Female	5256		50.46%
Body mass index	kg/m^2^	28.57	±	6.62
Hemoglobin A1C	%	5.5	±	0.6
Hemoglobin A1C	mmol/mol	37	±	6.6
Fasting plasma glucose	mmol/L	5.6	±	1.0
2-h postchallenged plasma glucose	mmol/L	6.6	±	2.8
Mexican Americans		1670		16.03%
Other Hispanics		1058		10.16%
Non-Hispanic Whites		4768		45.78%
Non-Hispanic Blacks		1884		18.09%
Others		1036		9.95%

Unweighted mean ± STD or *n* with percent.

**Table 2:** Comparison of the performance of the A1C ≥ 6.5% criterion to the fasting and 2-h postchallenged glucose criteria with subgroup analyses in subjects without a prior diagnosis of diabetes.

	Diabetes by FPG/2hPG	Total (*n*)	Diabetes by A1C		Diabetes by A1C				
			Unweighted		Weighted				
			*n*	%	%	Sensitivity	Specificity	Positive Predictive Value	Negative Predictive Value
						95% CI	95%CI	95%CI	95%CI
**a. All subjects**									
All	No	9607	53	0.55%	0.30%	25.90%	99.70%	84.07%	95.70%
	Yes	809	217	26.82%	25.90%	(25.87–25.92)	(99.70–99.70)	(84.03–84.10)	(95.70–95.70)
**b. Gender**									
Female	No	4877	23	0.47%	0.29%	24.52%	99.71%	83.34%	95.75%
	Yes	379	99	26.12%	24.52%	(24.49–24.56)	(99.71–99.71)	(83.29–83.40)	(96.75–95.76)
Male	No	4730	30	0.63%	0.31%	27.31%	99.69%	84.74%	95.64%
	Yes	430	118	27.44%	27.31%	(27.27–27.34)	(96.69–96.69)	(84.69–84.80)	(95.64–95.65)
**c. Race/ethnicity**									
Mexican American	No	1518	4	0.26%	0.21%	34.90%	99.79%	92.71%	95.18%
	Yes	152	45	29.61%	34.90%	(34.81–34.98)	(99.78–99.79)	(92.63–92.79)	(95.17–95.19)
Other Hispanic	No	966	8	0.83%	0.52%	27.38%	99.48%	76.58%	95.69%
	Yes	92	22	23.91%	27.38%	(27.27–27.49)	(99.48–99.49)	(76.40–76.75)	(95.68–96.70)
Non-Hispanic White	No	4380	8	0.18%	0.14%	21.35%	99.86%	90.62%	95.36%
	Yes	388	80	20.62%	21.35%	(21.32–21.38)	(99.86–99.86)	(90.58–90.66)	(95.35–95.36)
Non-Hispanic Black	No	1775	29	1.63%	1.30%	41.27%	98.70%	59.57%	97.31%
	Yes	109	44	40.37%	41.27%	(41.17–41.37)	(98.70–98.71)	(59.45–59.69)	(97.31–97.32)
Other	No	968	4	0.41%	0.23%	41.55%	99.77%	89.72%	97.29%
	Yes	68	26	38.24%	41.55%	(41.43–41.67)	(99.77–99.78)	(89.61–89.83)	(97.28–97.30)
**d. Age (years)**									
20–29	No	1860	0	0.00%	0.00%	14.32%	100.00%	100.00%	99.07%
	Yes	27	6	22.22%	14.32%	(14.22–14.43)	(100.00–100.00)	(100.00–100.00)	(99.07–99.08)
30–39	No	1867	2	0.11%	0.08%	36.21%	99.92%	91.94%	98.36%
	Yes	65	28	43.08%	36.21%	(36.12–36.31)	(99.92–99.92)	(91.85–92.02)	(98.35–98.36)
40–49	No	1846	7	0.38%	0.27%	29.38%	99.73%	83.13%	96.86%
	Yes	97	30	30.93%	29.38%	(29.31–29.45)	(99.73–99.73)	(83.03–83.22)	(96.86–96.87)
50–59	No	1526	12	0.79%	0.45%	32.57%	99.55%	82.99%	95.62%
	Yes	142	52	36.62%	32.57%	(32.51–32.63)	(99.55–99.55)	(82.91–83.07)	(95.61–95.63)
60–69	No	1293	17	1.31%	0.66%	29.81%	99.34%	83.14%	92.80%
	Yes	194	55	28.35%	29.81%	(29.75–29.87)	(99.33–99.34)	(83.06–83.22)	(92.79–92.81)
≥70	No	1215	15	1.23%	0.78%	15.81%	99.22%	81.44%	84.45%
	Yes	284	46	16.20%	15.81%	(15.77–15.85)	(99.21–99.22)	(81.35–81.54)	(84.43–84.46)
**e. BMI (kg/m^2^)**									
<22.50	No	1550	1	0.06%	0.03%	7.21%	99.97%	80.89%	98.21%
	Yes	51	6	11.76%	7.21%	(7.14–7.27)	(99.97–99.97)	(80.56–81.22)	(98.20–98.21)
22.50–24.99	No	1591	7	0.44%	0.15%	18.37%	99.85%	82.76%	96.88%
	Yes	93	17	18.28%	18.37%	(18.30–18.44)	(99.85–99.85)	(82.61–82.90)	(96.80–96.89)
25.00–27.49	No	1730	4	0.23%	0.09%	12.44%	99.91%	88.03%	95.64%
	Yes	137	22	16.06%	12.44%	(12.39–12.49)	(99.91–99.91)	(87.90–88.15)	(95.63–95.65)
27.50–29.99	No	1584	7	0.44%	0.29%	14.85%	99.71%	75.34%	95.09%
	Yes	142	29	20.42%	14.85%	(14.80–14.90)	(99.70–99.71)	(75.20–75.48)	(95.08–95.09)
30.00–32.49	No	1112	11	0.99%	0.41%	32.06%	99.59%	84.25%	95.59%
	Yes	106	33	31.13%	32.06%	(31.98–32.13)	(99.59–99.60)	(84.15–84.35)	(95.58–95.60)
32.50–34.99	No	758	1	0.13%	0.06%	34.48%	99.94%	97.68%	95.07%
	Yes	77	30	38.96%	34.48%	(34.39–34.56)	(99.93–99.94)	(97.64–97.73)	(95.06–95.08)
35.00–37.49	No	474	10	2.11%	1.50%	28.23%	98.50%	70.38%	91.55%
	Yes	75	20	26.67%	28.23%	(28.14–28.31)	(98.49–98.50)	(70.25–70.51)	(91.53–91.57)
≥37.50	No	808	12	1.49%	0.92%	46.02%	99.08%	87.01%	93.18%
	Yes	128	60	46.88%	46.02%	(45.95–46.08)	(99.07–99.08)	(86.95–87.07)	(93.17–93.19)

**Table 3:** Clinical characteristics by the diagnostic categories in subjects without a prior diagnosis of diabetes.

		Non-Diabetic by Both A1C and FPG/2hPG		Diabetic by FPG/2hPG but not A1C		Diabetic by FPG/2hPG and A1C		Diabetic by A1C But Not FPG/2hPG		*p* *
*n* (unweighted percent)		9554	91.72%	592	5.68%	217	2.08%	53	0.51%	
weighted percent			94.02%		4.22%		1.48%		0.28%	
Age	Years	45	(45–45)	62	(59–61)	56	(54–58)	62	(55–65)	0.01
Gender (weighted percent)	Female		52.23%		51.57%		47.95%		50.57%	<0.0001
Body mass index	kg/m^2^	28.30	(28.17–28.43)	30.19	(30.06–31.17)	33.45	(33.62–35.49)	33.99	(32.58–36.87)	<0.0001
Race/ethnicity (weighted percent)										<0.0001
Mexican Americans			8.22%		9.27%		14.22%		5.90%	
Other Hispanics			5.41%		5.43%		5.86%		9.45%	
Non-Hispanic Whites			68.54%		74.30%		57.71%		31.51%	
Non-Hispanic Blacks			10.80%		6.64%		13.34%		47.78%	
Others			7.02%		4.36%		8.87%		5.36%	
Hemoglobin A1C	%	5.4	(5.3–5.4)	5.8	(5.7–5.8)	7.8	(7.6–7.8)	6.6	(6.3–6.9)	<0.0001
Hemoglobin A1C	mmol/mol	36	(34–36)	40	(39–40)	62	(60–62)	49	(45–52)	<0.0001
FPG	mmol/L	5.4	(5.4–5.4)	6.7	(6.5–6.8)	9.5	(9.2–9.7)	6.3	(5.7–6.7)	<0.0001
2hPG	mmol/L	5.9	(5.9–5.9)	11.7	(11.5–12.0)	17.0	(16.3–17.2)	8.2	(7.2–9.2)	<0.0001
FPG ≥ 7.0 mmol/L only			-		21.37%		6.62%		-	<0.0001
2hPG ≥ 11.1 mmol/L only			-		59.72%		14.16%		-	
Both FPG ≥ 7.0 mmol/L and 2hPG ≥ 11.1 mmol/L			-		18.90%		79.22%		-	

Weighted mean (95% CI). * Weighted *p* by ANOVA for 3 diabetic groups.

**Table 4:** Weighted prevalence of newly defined diabetes by A1C, sensitivity, specificity, positive predictive value, negative predictive value with 95% confidence intervals, and the area under ROC curve based on different A1C criteria in comparison to the fasting and 2-h postchallenged plasma glucose criteria in subjects without a prior diagnosis of diabetes.

	Prevalence of Newly Defined Diabetes by A1c	Sensitivity	Specificity	Positive Predictive Value	Negative Predictive Value	Area under ROC
A1C ≥ 5.5% (37 mmol/mol)	37.11%	84.47%	60.64%	11.48%	98.48%	0.8353
	(37.11–37.12)	(84.45–84.49)	(60.64–60.65)	(11.48–11.49)	(98.47–98.48)	
A1C ≥ 5.6% (38 mmol/mol)	27.68%	78.84%	70.64%	13.96%	98.22%	0.8289
	(27.68–27.69)	(78.81–78.86)	(70.63–70.65)	(13.95–13.97)	(98.22–98.22)	
A1C ≥ 5.7% (39 mmol/mol)	19.27%	73.00%	79.56%	17.76%	97.99%	0.8251
	(19.27–19.28)	(72.97–73.02)	(79.56–79.57)	(17.74–17.77)	(97.99–97.99)	
A1C ≥ 5.8% (40 mmol/mol)	12.96%	65.24%	86.25%	22.29%	97.62%	0.8222
	(12.96–12.97)	(65.21–65.27)	(86.25–86.26)	(22.28–22.31)	(97.62–97.62)	
A1C ≥ 5.9% (41 mmol/mol)	8.39%	58.62%	91.11%	28.49%	97.33%	0.8162
	(8.38–8.39)	(58.59–58.64)	(91.10–91.11)	(28.47–28.51)	(97.33–97.33)	
A1C ≥ 6.0% (42 mmol/mol)	5.23%	51.75%	94.46%	36.08%	97.00%	0.8108
	(5.22–5.23)	(51.72–51.78)	(94.45–94.46)	(36.057–36.10)	(97.00–97.01)	
A1C ≥ 6.1% (43 mmol/mol)	3.15%	45.44%	96.66%	45.14%	96.70%	0.8055
	(3.15–3.15)	(45.41–45.47)	(96.66–96.66)	(45.11–45.17)	(96.70–96.70)	
A1C ≥ 6.2% (44 mmol/mol)	1.73%	40.69%	98.17%	57.31%	96.48%	0.8047
	(1.73–1.73)	(40.66–40.71)	(98.17–98.17)	(57.27–57.34)	(96.47–96.48)	
A1C ≥ 6.3% (45 mmol/mol)	0.95%	36.28%	99.00%	68.62%	96.26%	0.8034
	0.94–0.95)	(36.25–36.31)	(99.00–99.00)	(68.58–68.65)	(96.25–96.26)	
A1C ≥ 6.4% (46 mmol/mol)	0.53%	30.14%	99.43%	76.29%	95.93%	0.7994
	(0.53–0.53)	(30.11–30.17)	(99.43–99.43)	(76.25–76.33)	(95.92–95.93)	

**Table 5:** Prevalence of diabetic retinopathy based on A1C categories in subjects with established diabetes.

A1C	Unweighted			Weighted	
	Total	Retinopathy		Retinopathy	
	*n*	*n*	%	%	95% CI
≤5.5 (37 mmol/mol)	251	38	15.14%	13.97%	(13.91–14.03)
5.6–5.8 (38–40 mmol/mol)	283	38	13.43%	13.10%	(13.05–13.15)
5.9–6.0 (41–42 mmol/mol)	253	36	14.23%	14.46%	(14.40–14.52)
6.1–6.2 (43–44 mmol/mol)	245	49	20.00%	13.05%	(13.00–13.11)
6.3–6.4 (45–46 mmol/mol)	301	33	10.96%	11.52%	(11.47–11.57)
6.5–6.6 (48–49 mmol/mol)	257	52	20.23%	18.32%	(18.25–18.38)
6.7–6.8 (50–51 mmol/mol)	256	59	23.05%	18.92%	(18.85–18.98)
6.9–7.0 (52–53 mmol/mol)	227	41	18.06%	20.93%	(20.85–21.01)
7.1–7.3 (54–56 mmol/mol)	295	62	21.02%	18.69%	(18.63–18.75)
7.4–7.6 (57–60 mmol/mol)	249	60	24.10%	21.57%	(21.50–21.64)
7.7–8.0 (61–64 mmol/mol)	265	61	23.02%	21.11%	(21.03–21.18)
8.1–8.6 (65–70 mmol/mol)	270	77	28.52%	26.33%	(26.26–26.41)
8.7–9.5 (72–80 mmol/mol)	260	73	28.08%	23.22%	(23.14–23.29)
9.6–10.9 (81–96 mmol/mol)	244	76	31.15%	27.57%	(27.49–27.65)
≥11.0 (97 mmol/mol)	251	76	30.28%	31.52%	(31.43–31.61)

## Data Availability

All data analyzed in this study are obtained from and available at the website of the National Health and Nutrition Examination Survey, Centers for Disease Control and Prevention (https://wwwn.cdc.gov/nchs/nhanes/Default.aspx (accessed on 20 March 2020).
